# Three-dimensional printing models in congenital heart disease education for medical students: a controlled comparative study

**DOI:** 10.1186/s12909-018-1293-0

**Published:** 2018-08-02

**Authors:** Wei Su, Yunbin Xiao, Siping He, Peng Huang, Xicheng Deng

**Affiliations:** 1 Research Unit for Pediatrics, Xiangnan University School of Medicine, Chenzhou, 423000 China; 2 Heart Center, Hunan Children’s Hospital, No. 86 Ziyuan Road, Changsha, 410007 China; 3 Department of Radiology, Hunan Children’s Hospital, Changsha, 410007 China

**Keywords:** Medical education, Three-dimensional printing, Congenital heart disease

## Abstract

**Background:**

This study sought to assess, using subjective (self-assessment) and objective (MCQ) methods, the efficacy of using heart models with ventricular septal defect lesions produced with three-dimensional printing technology in a congenital heart disease curriculum for medical students.

**Methods:**

Three computed tomography datasets of three subtypes of ventricular septal defects (perimembranous, subarterial and muscular, one for each) were obtained and processed for building into and printing out 3D models. Then a total of 63 medical students in one class were randomly allocated to two groups (32 students in the experimental, and 31 the control). The two groups participated in a seminar with or without a 3D heart model, respectively. Assessment of this curriculum was carried out using Likert-type questionnaires as well as an objective multiple choice question test assessing both knowledge acquisition, and structural conceptualization. Open-ended questions were also provided for getting advice and suggestion on 3D model utilization in CHD education.

**Results:**

With these 3D models, feedback shown in the questionnaires from students in experimental group was significantly more positive than their classmates in the control. And the test results also showed a significant difference in structural conceptualization in favor of the experimental group.

**Conclusion:**

It is effective to use heart models created using current 3D printing technology for congenital heart disease education. It stimulates students’ interest in congenital heart disease and improves the outcomes of medical education.

**Electronic supplementary material:**

The online version of this article (10.1186/s12909-018-1293-0) contains supplementary material, which is available to authorized users.

## Background

Three-dimensional printing, a kind of rapid prototyping and manufacturing technology, has been widely utilized in medicine [1–3]. Applications of 3D printing technology in the medical field includes surgical guidance during surgery, preoperative procedural planning [4–7] as well as management of difficult clinical situations [8]. Medical education has also witnessed its wide application [9–12]. Simulation-based training and education with 3D models for students (including nursing, nursing anesthesia, and medical trainees) has been reported to improve understanding of medical knowledge and clinical outcomes [13–15].

Congenital heart defects (CHD) is a group of common defects with a prevalence of 0.8–1.2% of all live birth [16]. A clinical understanding of CHD is crucial as many of the anomalies can be life threatening. The traditional educational approach to teaching CHD involves the use of pathological specimens, off-the-shelf normal heart anatomic models made of plastics, medical imaging data(including CT, MR and Echocardiography), as well as textbooks. This approach is difficult to conceptualize and visualize for medical students considering the variability of all the different types of CHDs [17]. Since every single CHD case is unique and different in terms of their anatomy and pathophysiology, this poses a huge challenge for learners.

The recent advancement and popularization of three-dimensional (3D) printing has made it possible to create high-fidelity heart models with complex cardiac lesions from source imaging data [18]. Theoretically, 3D printed models have better spatial and structural visualization and can be used as didactic tools for better understanding of complex heart or vessel anatomy as well as easier explanation of abnormal anatomical heart and vessel structures. Some previous studies have shown its effectiveness of using 3D-printed models in CHD curriculum [11, 18, 19]. However, no controlled study with objective evaluation methods has ever been performed. The non-randomized controlled nature and subjective assessment in previous studies made the results less convincing. Therefore, we carried out this randomized controlled study to verify the efficacy of 3D printed heart model use in medical education.

## Methods

**Fig. 1:**
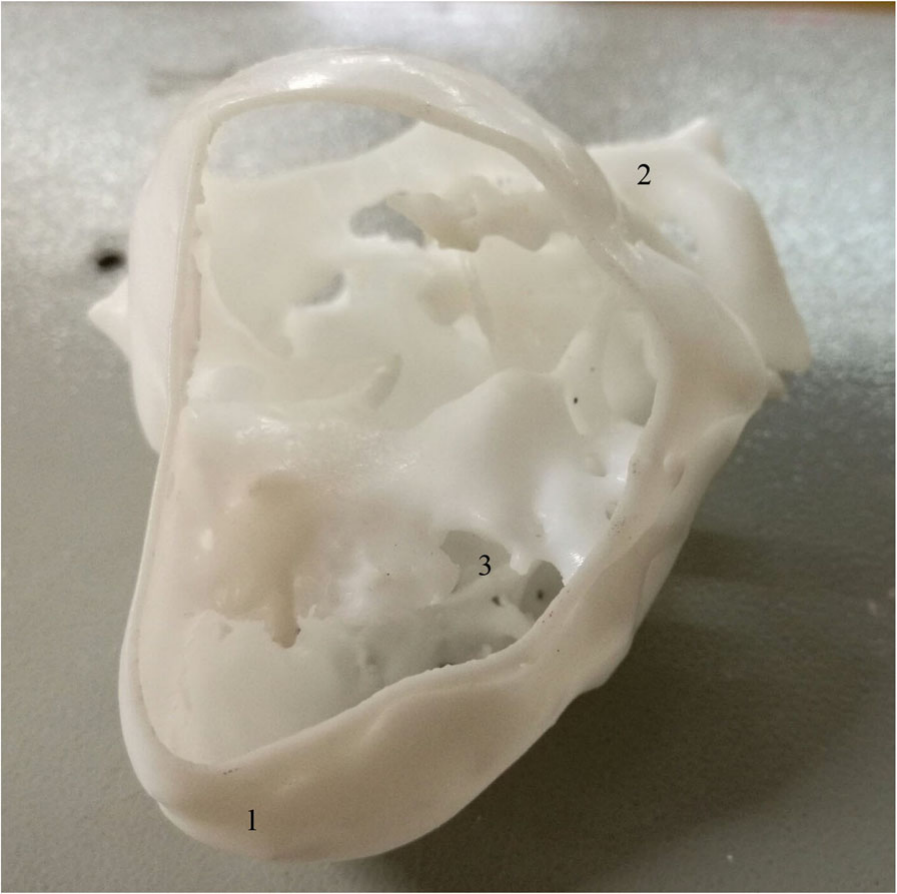
Front view of a model. 1. apex; 2. aortic arch; 3. ventricular septal defect

**Fig. 2:**
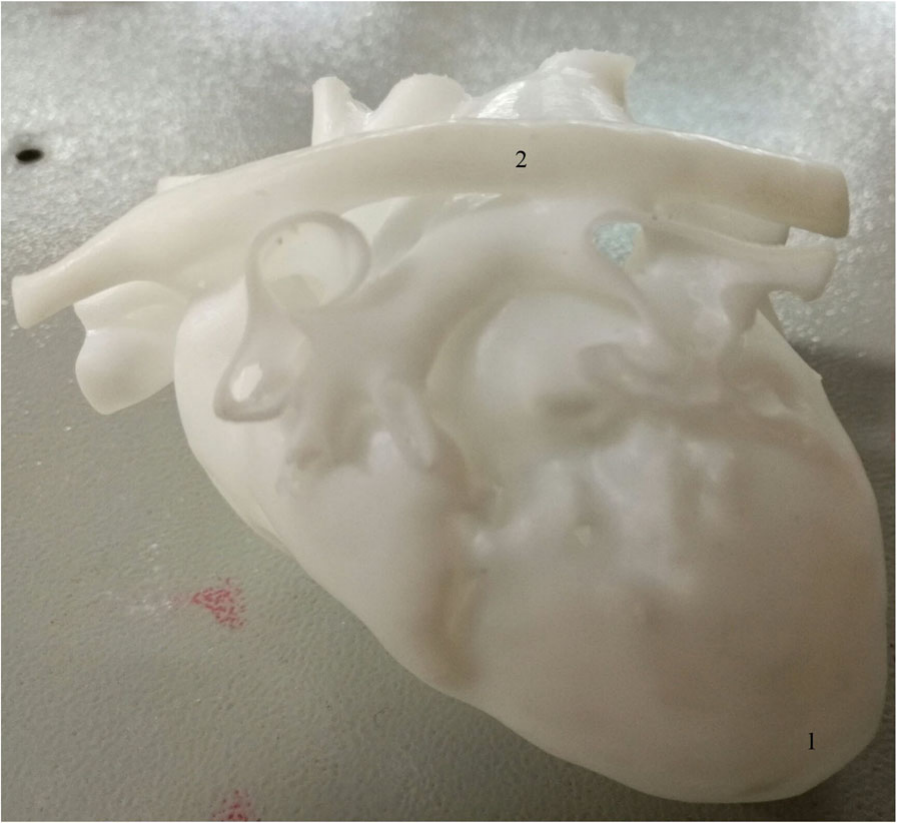
Back view of the same model as shown in Fig. 1. 1. apex; 2. descending aorta

Computed tomography imaging (CT) data were extracted and 3D printing process was performed to produce lesion-specific models. In detail, CT data of three ventricular septal defects (VSD) (subarterial, membranous, and muscular types, one for each) were exported from institutional image archive system and then imported and analyzed. After post-process, the reconstructed images underwent 3D volume rendering to create a digital heart model. Following optimization and simplification, final data were generated and sent to SL600 3D printer (ZRapid Tech, Wujiang, China) for model printing. High-fidelity plastic heart models were successfully produced one for each of three common ventricular septal defect subtypes (subarterial, membranous, and muscular types) (Figs. 1 and 2).

Curriculum incorporating these 3D heart models was then developed. Besides the 3D prints, compulsory CHD syllabus involving relevant knowledge on anatomy and classification, pathophysiology, clinical findings, workups, clinical management and prognosis of ventricular septal defects for medical students was included in the curriculum and then used in seminars. The curriculum developer, who was also the lecturer to hold the seminars, was an associate professor of pediatrics with more than 10 years of teaching experience of lectures and seminars for medical students and medical graduates.

A class of 63 medical students was chosen to carry out the study. They were informed beforehand about this study and agreed to participate. They were randomly allocated to two groups, 32 students in the experimental and 31 in the control, using computerized random number generation method. All the subjects participated in one of two teaching seminars by the same lecturer. The inter-group demographics were compared and showed no statistical difference. Instead of pre-seminar assessment, we used latest academic test score as baseline academic performance (Table 1).

**Table 1:** Intergroup comparison of demographics and academic performance

Group demographics	Experimental	Control	*P* Value
Gender(M/F) (total number)	16/16 (32)	14/17 (31)	0.802
Age	21.00 ± 0.57	21.06 ± 0.58	0.655
Academic performance	73.43 ± 10.84	76.68 ± 10.93	0.242

Seminars were designed and structured for the two groups. For the experimental group, the seminar consisted of two consecutive components: (1) a succinct introduction to 3D printing technology and its use in medical education and practice; (2) a didactic session on ventricular defects integrating 3D models in teaching subtypes of VSD, specifically, in its classification and pathophysiology. Yet for the control subjects, they attended a seminar with only the second component but without 3D models (only with images and animations) (Table 2). The teaching method and curriculum in the control group had been used for years and approved by teachers and students, so the participants in control group were not regarded as being at a disadvantage of learning.

**Table 2:** VSD cirriculum in the two groups

Cirriculum structure	Experimental(minutes)	Control(minutes)
Introduction to 3D printing technology	5	N/A
Anatomy and classification	5(3D prints used)	5
Pathophysiology	15(3D prints used)	15
Clinical findings	3	3
Workups	3	3
Clinical management and prognosis	4	4
Total seminar duration	35	30
After-seminar test and questionaire	25	25

At the end of each seminar, all participants completed a subjective 1–10 scale Likert-type questionnaire and a prepared objective multiple choice tests (MCQ). The questionnaires were identical between the two groups. The questionnaire included 10 items that examined two main educational components which were knowledge acquisition (including clinical presentations and pathophysiology, 3 items) and structural conceptualization (including classification/types and anatomy, 2 items) as well as 5 items regarding students’ appraisal of the seminars and 3D prints. Each item got a score ranging 1–10 and the total score of the two main educational components for each participant were summed up. The MCQs included 10 items on the anatomy, pathophysiology and scenario cases of VSD. The same MCQs had been used as standard test after didactic session for VSD in the late several years before the study to assess the students’ grasp of knowledge about CHD. The questionnaire as well as the MCQ was developed by one of the authors who was the only teacher involved in the seminars. The MCQs and questionnaire papers were marked with personal information for recording each student’s academic performance. Then the data were de-identified for use in this study. All original documents were kept on file in case of a review.

Data was statistically presented and processed as appropriate. Shapiro-Wilk test was used to confirm if it was normally distributed where necessary. Statistical analysis was then conducted using the Chi-square and Student’s T test as appropriate; two-tailed *P* values of 0.05 were used as indicators of significance. Two open-ended questions were also provided in the experimental group for advice on 3D model utilization in CHD education in order for improvement in the future. The focuses of the answers were extracted manually and accumulated as frequencies. The original version and translated MCQ paper, questionnaire and open-ended questions have been uploaded as Additional files 1, 2, 3.

## Results

There was no difference in gender, age and academic performance between the groups (Table 1). At the end of each seminar, the questionnaire results showed that students in the experimental group reported significant improvement in VSD learning and better seminar outcomes (*P* < .0001) (Table 3). The test results were statistically significant in favor of the experimental group (*P* = 0.02). The inter-group differences of both questionnaire and test results were statistically significant in structural conceptualization (*P* = 0.02, 0.03, respectively), but not in knowledge acquisition (*P* = 0.09, 0.06, respectively).

**Table 3:** Tests and questionnaire results

Test / appraisal(score)^a^	Experimental	Control	*P* Value
Test score(100)	62.50 ± 19.04	51.29 ± 17.55	0.02
Structural conceptualization (30)	18.44 ± 6.67	14.52 ± 7.11	0.03
Knowledge acquisition (70)	44.06 ± 16.37	36.77 ± 12.54	0.06
Likert-type questionnaire (100)^b^	72.19 ± 14.91	56.12 ± 10.55	< 0.0001
Structural conceptualization (20)	15.50 ± 2.22	14.16 ± 2.17	0.02
Knowledge acquisition (30)	22.63 ± 3.00	21.39 ± 2.50	0.09

^a^ each item has a full score of 10

^b^ the questionnaire contains other 5 items that are not included in the table

Data from open-ended questions included in the questionnaires in experimental group showed advantages of using 3D models over traditional teaching method and aspects that need to be improved. On the one hand, a total of 27(84.4%) students reported better understanding of heart anatomy, followed by 16(50.0%) admitted a positive interest in cardiology and cardiac surgery as show in the results of question 1. On the other hand, in question 2, as high as 18(56.2%) subjects reported a need for improvement in cardiac structures especially intra-cardiac ones exemplified by the valves and their apparatuses in the 3D prints. Similar to that was a complaint about lack of explicit delineation of structures by 11(34.4%) students.

## Discussion

In the present study, our finding has shown overall improvement and better structural conceptualization in sub-analysis with both test scores and questionnaire following a 3D heart model assisted seminar.

The major difference of this study from previous ones [18] is both MCQ and self-assessment were used in the evaluation of the simulation based teaching and a randomized controlled study designed was used. In previous studies [20], the subjective methods used may have potential statistical bias and warranted a case-controlled study. While our result confirmed the conclusion from previous studies, the comparative nature of this study made it more convincing, though we used previous academic test score as a baseline instead of pre-seminar test. We did not use MCQs before the seminars for fear that it may bring bias if the same test was used both before and after the seminars. According to our results, the students had no inter-group difference in academic performance. (Table 1).

Though every single case with CHD is regarded unique, we only printed out three models representing different types of VSD. First, this is a pilot study to verify it’s efficacy in medical student education. Second, for medical education purpose, we thought it was enough to use three typical models to convey sufficient information to students, rather than in a clinical setting that every single case might seem different with regard to anatomy of a VSD.

As for the 3D printing technology and the imaging source and resolution of printed models, though some specific structures especially intra-cardiac ones, including heart valves and trabecular muscles, needed to be improved in fidelity, the CT dataset proved to be a sufficient source to create ventricular septal defect models with the aid of relevant softwares and 3D printing technology [21]. The shortcomings in detailing intra-cardiac anatomy are not because of limitation of the 3D printing per se, but for limited resolution of source images. Computed tomography (CT) images and cardiac magnetic resonance imaging (MRI) were used as source data for printing the high-fidelity models. Compared to MRI, CT provides superior spatial resolution [22] but radiation is an inevitable shortage. Both of them as image source are limited and some researchers [11] have developed an approach to utilizing echocardiographic images as source data to enhance fidelity of heart models, especially for valve anatomy.

The use of 3D productions in medical education have several advantages: first, comparing to cadaver, it is much cheaper which may in turn alleviate the financial, ethical, cultural, and logistical difficulties of maintaining a cadaver-based curriculum [12]; second, comparing to pictorial images, diagrams, and conventional echocardiography, which are all two-dimensional hence conceptually challenging for beginning learners, It gives a true spatial relationship to allow tangible manipulation of the extra- and intra-cardiac structures [23]. Third, comparing to anatomical off-the-shelf models, even those of high quality are rather schematic and do not show the range of variation present in different human populations in health and disease [24, 25]. The anatomical advantage of using 3D prints was confirmed by our results in both test and questionnaire. Also, the results from open-ended questions showed an increased interest of the students in cardiology and cardiac surgery.

However, there were some limitations in the study. First, for the statistical aspects, the subject number was relatively small and randomization was undertaken in a preexisting class. And we did not test the students prior to the seminar. Instead, we used a latest academic score for baseline comparison. As such, there may remain some biases in the study. Second, as mentioned before, these 3D prints are not without drawbacks. Currently, limited to image quality, it remains a challenge to accurately print out intra-cardiac structures, making its application confined to specific types of CHD.

As mentioned above, 3D printed models are generally regarded to advantage over other media in medical education for its superior spatial and structural visualization and patient-specific features. This is also demonstrated by the present study results, especially better structural conceptualization in sub-analysis results. Further application in CHD education may include multiple disease including atrial septal defect, tetralogy of Fallot, etc. In the near future, 3D printing technologies are expected to advance to make more accurate heart prints, especially intra-cardiac structures including valves and their apparatus.

## Conclusions

In this study we have demonstrated the efficacy of incorporating a 3D printing heart model into a medical curriculum about CHD. We have used both MCQ and self-assessment methods in this comparative study and demonstrated that better structural conceptualization by students were achieved. As 3D printing continues to advance and simulation-based education becomes more extensively utilized, this novel technology is expected to broadly apply in the education of congenital heart defects.

## Additional files

Additional file 1: Questionnaire and quiz scores. (XLSX 13 kb)

Additional file 2: Quiz MCQs and translated stems. (DOC 29 kb)

Additional file 3: Original questionnaires and English translation. (DOC 27 kb)

## Abbreviations

3D: Three dimensional
CHD: Congenital heart defects
CT: Computed tomography imaging
MCQ: Multiple choice question/test
MRI: Magnetic resonance imaging
VSD: Ventricular septal defect
