# Supplementary material for: Three-dimensional printing models in congenital heart disease education for medical students: a controlled comparative study
Source: BMC Med Educ. 2018 Aug 2;18:178. doi: 10.1186/s12909-018-1293-0 (PMC6090870; doi:10.1186/s12909-018-1293-0)
Supplement: Supplementary file 2 — Quiz MCQs and translated stems. (DOC 29 kb) [file 12909_2018_1293_MOESM2_ESM.doc]

**随堂测验**

1、先心病中最常见的类型是: （ ）

A、 室间隔缺损 B、 房间隔缺损

C、 动脉导管未闭 D、 法乐四联症

E、 肺动脉狭窄

2、室间隔缺损中最常见类型为: （ ）

A、膜周 B、流入道

C、肌部 D、干下

E、嵴内

3、哪种类型室间隔缺损中解剖上靠近肺动脉瓣: （ ）

A、膜周 B、流入道

C、肌部 D、干下

E、嵴内

4、室间隔缺损的杂音特点为:（ ）

A、胸骨左缘第2肋间连续性机器样杂音

B、分流量大者心尖区有较短的舒张期杂音

C、胸骨左缘第3-4肋间收缩期杂音

D、杂音随肺循环压力增高而增强

E、肺动脉瓣区第二音增强，但多被杂音淹没而不易识别

5、室间隔缺损可引起下列部位负荷增加，除外:( )

A、右心房 B、右心室

C、肺循环 D、左心房

E、左心室

6、以下是室间隔缺损的临床体征，但应除外( )

A、心界扩大 B、杂音最响处可触及收缩期震颤

C、杂音传导广泛 D、P2固定分裂

E、心尖搏动弥散

7、左向右分流型先心病可出现以下症状但除外：（ ）

A、体循环血量减少 B、肺循环血量增多

C、肺动脉压力增高 D、肺动脉压力下降

E、反复肺炎

8、室间隔缺损患儿，杂音逐渐减弱，P2亢进，可能是：
A、分流量大 B、合并房间隔缺损
C、Roger病 D、肺动脉高压
E、以上都不是

9、哪一项不是室间隔缺损的并发症：
A、肺炎 B、心力衰竭
C、缺氧发作 D、感染性心内膜炎
E、肺水肿

10、患儿3岁，胸骨左缘第3--4肋间可闻Ⅲ级粗糙全收缩期杂音，于杂音最响处可触及收缩期震颤，心尖部可闻舒张期杂音，肺动脉瓣区第2心音亢进，应考虑：
 A．室间隔缺损(Roger病) B．大型室间隔缺损 C．房间隔缺损
 D．动脉导管未闭  E．法洛四联症

**Translated stems:**

1, the most common type of congenital heart disease is: ()

2, the most common type of ventricular septal defect: ()

3,which type of ventricular septal defect is anatomically close to pulmonary valve:()

4, the characteristic murmur of ventricular septal defects: ()

5, ventricular septal defect can cause the following overload, with the exception of: ()

6, the following is the clinical signs of ventricular septal defect, except for ()

7, the following symptoms can occur in left to right shunt congenital heart disease, except: ()

8,in a child with ventricular septal defect, the heart murmur gradually weakened with Increased

intensity of P2. The cause may be:()

9,which is not a complication of ventricular septal defect:()

10,In a 3-year-old children, grade Ⅲ rough pansystolic murmur can be heard at LSB ICS3-4. And systolic tremor can be felt where the murmur is most intensive, a diastolic murmur can be heard at apex, with increase P2. The working diagnosis is()
