# Supplementary material for: Three-dimensional printing models in congenital heart disease education for medical students: a controlled comparative study
Source: BMC Med Educ. 2018 Aug 2;18:178. doi: 10.1186/s12909-018-1293-0 (PMC6090870; doi:10.1186/s12909-018-1293-0)
Supplement: Supplementary file 3 — Original questionnaires and English translation. (DOC 27 kb) [file 12909_2018_1293_MOESM3_ESM.doc]

**课堂教学评价表**

1. 通过本节课学习，你认为对心脏结构的掌握评几分？(10分代表非常好，1分代表不好)

1 2 3 4 5 6 7 8 9 10

1. 通过本节课学习，你认为对先天性心脏病知识的掌握评几分？(10分代表非常好，1分代表不好)

1 2 3 4 5 6 7 8 9 10

3、通过本节课学习，你认为对室间隔缺损知识的掌握评几分？(10分代表非常好，1分代表不好)

1 2 3 4 5 6 7 8 9 10

4、通过本节课学习，你认为教师讲述室间隔缺损的能力评几分？(10分代表非常好，1分代表不好)

1 2 3 4 5 6 7 8 9 10

5、你对3D打印进程的认知能力评几分？(10分代表非常了解，1分代表不了解)

1 2 3 4 5 6 7 8 9 10

6、通过本节课学习，你认为对室间隔缺损类型掌握评几分？(10分代表非常好，1分代表不好)

1 2 3 4 5 6 7 8 9 10

7、通过本节课学习，你认为对室间隔缺损治疗方法的掌握评几分？(10分代表非常好，1分代表不好)

1 2 3 4 5 6 7 8 9 10

8、你对本次课堂教学效果的评价？(10分代表非常好，1分代表不好)

1 2 3 4 5 6 7 8 9 10

9、你认为3D打印模型对课堂教学效果提升的评价? (10分代表非常好，1分代表不好)

1 2 3 4 5 6 7 8 9 10

10、你认为将3D打印模型引入临床技能教学，以提升技能教学效果的可能性？(10分代表非常可能，1分代表不可能)

1 2 3 4 5 6 7 8 9 10

开放问题：

1.你认为3d模型对你先心病学习最大的帮助在哪方面？

2.你认为先心病3d模型有什么不足和需要改进的地方？

**Translated in English**

1, after this seminar, how do you scale your grasp of the heart structure? (10 points for very good, 1 point for very poor)

1 2 3 4 5 6 7 8 9 10

2, after this seminar, how do you scale your knowledge of congenital heart disease? (10 points for very good, 1 point for very poor)

1 2 3 4 5 6 7 8 9 10

3, after this seminar, how do you scale your grasp of the ventricular septal defect knowledge? (10 points for very good, 1 point for very poor)

1 2 3 4 5 6 7 8 9 10

4, after this seminar, how do you think of the teacher’s didactic ability of ventricular septal defect? (10 points for very good, 1 point for very poor)

1 2 3 4 5 6 7 8 9 10

5, how do you scale your understanding of 3d printing process in medical application? (10 points for very good, 1 point for very poor)

1 2 3 4 5 6 7 8 9 10

6, after this seminar, how do you scale your grasp of the types of ventricular septal defect? (10 points for very good, 1 point for very poor)

1 2 3 4 5 6 7 8 9 10

7, after this seminar, how do you scale your grasp of the treatment of ventricular septal? (10 points for very good, 1 point for very poor)

1 2 3 4 5 6 7 8 9 10

8, how do you scale your evaluation of the effectiveness of this seminar? (10 points for very good, 1 point for very poor)

1 2 3 4 5 6 7 8 9 10

9, do you think 3d printing model has improved the effectiveness of the seminar? (10 points for very good, 1 point for very poor)

1 2 3 4 5 6 7 8 9 10

10, how do you think the possibility to enhance teaching effectiveness by introducing the 3d printing model into the clinical skills teaching? (10 points on behalf of very likely, 1 minute on behalf of impossible)

1 2 3 4 5 6 7 8 9 10

Open-ended questions

1.what do you think is most helped in learning congenital heart disease when using a 3d heart model？

2.what shortage that need to be improved do you think the model has？
